# Resource Partitioning between Bacteria, Fungi, and Protists in the Detritusphere of an Agricultural Soil

**DOI:** 10.3389/fmicb.2016.01524

**Published:** 2016-09-26

**Authors:** Susanne Kramer, Dörte Dibbern, Julia Moll, Maike Huenninghaus, Robert Koller, Dirk Krueger, Sven Marhan, Tim Urich, Tesfaye Wubet, Michael Bonkowski, François Buscot, Tillmann Lueders, Ellen Kandeler

**Affiliations:** ^1^Institute of Soil Science and Land Evaluation, University of HohenheimStuttgart, Germany; ^2^Institute of Groundwater Ecology, Helmholtz Zentrum München - German Research Center for Environmental HealthNeuherberg, Germany; ^3^Department of Soil Ecology, Helmholtz Centre for Environmental Research Leipzig-HalleHalle, Germany; ^4^Institute of Biology, University of LeipzigLeipzig, Germany; ^5^Department of Terrestrial Ecology, Institute of Zoology, University of CologneKöln, Germany; ^6^Department of Bacterial Physiology, Institute for Microbiology, Ernst-Moritz-Arndt University of GreifswaldGreifswald, Germany; ^7^German Centre for Integrative Biodiversity Research (iDiv)Leipzig, Germany

**Keywords:** plant litter decomposition, rRNA-SIP, stable isotope probing, amplicon sequencing, energy channels

## Abstract

The flow of plant-derived carbon in soil is a key component of global carbon cycling. Conceptual models of trophic carbon fluxes in soil have assumed separate bacterial and fungal energy channels in the detritusphere, controlled by both substrate complexity and recalcitrance. However, detailed understanding of the key populations involved and niche-partitioning between them is limited. Here, a microcosm experiment was performed to trace the flow of detritusphere C from substrate analogs (glucose, cellulose) and plant biomass amendments (maize leaves, roots) in an agricultural soil. Carbon flow was traced by rRNA stable isotope probing and amplicon sequencing across three microbial kingdoms. Distinct lineages within the *Actinobacteria, Bacteroidetes, Gammaproteobacteria, Basidiomycota, Ascomycota* as well as *Peronosporomycetes* were identified as important primary substrate consumers. A dynamic succession of primary consumers was observed especially in the cellulose treatments, but also in plant amendments over time. While intra-kingdom niche partitioning was clearly observed, distinct bacterial and fungal energy channels were not apparent. Furthermore, while the diversity of primary substrate consumers did not notably increase with substrate complexity, consumer succession and secondary trophic links to bacterivorous and fungivorous microbes resulted in increased food web complexity in the more recalcitrant substrates. This suggests that rather than substrate-defined energy channels, consumer succession as well as intra- and inter-kingdom cross-feeding should be considered as mechanisms supporting food web complexity in the detritusphere.

## Introduction

Microbes perform many crucial functions in soil, such as the primary decomposition of plant litter and the turnover of soil organic matter (Berg and McClaugherty, 2008; Schmidt et al., 2011). Despite the importance of these processes for global carbon budgets, factors controlling the roles and activities of distinct soil microbiota in the detritusphere are still poorly understood. Bacteria are classically thought to be more active in the degradation of labile organic matter as well as in early phases of litter decomposition (“bacterial energy channel”). In contrast, fungi are assumed to be more important in the degradation of complex and recalcitrant substrates, as well as in later stages of litter decomposition (“fungal energy channel”; de Boer et al., 2005; Paterson et al., 2008). However, recent work suggests that the role of bacteria in the mineralization of recalcitrant substrates as well as that of fungi in early stages of litter decomposition may also be significant (Bastian et al., 2009; Poll et al., 2010; España et al., 2011). This has fueled an ongoing debate on the specific roles and quantitative contributions of distinct soil microbiota in overall litter degradation (Strickland and Rousk, 2010; Rousk and Frey, 2015).

Resource partitioning between microbes consuming complex substrates is also considered an important driver of soil microbial diversity (Zhou et al., 2002). This has been demonstrated for primary substrate degraders in soil (Goldfarb et al., 2011), but can also be assumed for higher trophic levels, i.e., that of protists. These are known to exert important top-down controls on the bacterial energy channel (Ekelund and Ronn, 1994), but can also harbor primary detritus decomposers (Termorshuizen and Jeger, 2008). This complicates the quantitative understanding of their role in detritusphere energy channels. Protistan grazing on fungal hyphae and yeasts (Adl and Gupta, 2006) may represent an under-investigated cross-link between both energy channels. Hence, niche partitioning and the contributions of distinct key populations to detritusphere carbon flow across microbial kingdoms remains a matter of investigation.

The amendment of isotopically labeled substrates is an important experimental approach to tackle such questions. Especially in combination with nucleic acid-based stable isotope probing (SIP), this strategy makes it possible to trace substrate-derived carbon flow and to identify key populations involved. A number of studies have used ^13^C-labeled detritusphere substrate analogs such as cellulose or derived di- and monosaccharides to follow their uptake by soil bacteria, fungi, and/or protists (Haichar et al., 2007; Schellenberger et al., 2010; Eichorst and Kuske, 2012; Štursová et al., 2012; Chatzinotas et al., 2013; Verastegui et al., 2014; Wang et al., 2015; Pepe-Ranney et al., 2016). However, few studies have addressed the degradation of actual plant biomass by soil bacteria, e.g., with labeled residues of wheat (Bernard et al., 2007, 2009), maize (Fan et al., 2014), or rice root callus (Li et al., 2011). Carbon flow from the latter has been probed up to the levels of fungi and protists (Murase et al., 2012). Results of this relatively small number of studies suggest that the partitioning of natural detritusphere substrates between microbial kingdoms in soil warrants further elaboration.

Here, a SIP microcosm experiment with soil from an experimental maize field (Kramer et al., 2012; Scharroba et al., 2012) is reported. The site, dominated by Cambisols in conjunction with Luvisols, represents one of the most common and productive agricultural soil types in Central Europe (Akça et al., 2005). Treatments included the amendment of U-^13^C-labeled glucose and cellulose as defined mono- and polymeric substrates, and U-^13^C-maize leaves and roots as complex detritusphere substrates. Key label-assimilating bacteria, fungi, and protists were traced by rRNA-SIP at early and later stages of decomposition. We hypothesized that (i) distinct bacterial and fungal substrate utilization channels could be identified, (ii) the number of primary substrate consumers should increase with substrate complexity, and (iii) niche partitioning and succession should influence the relative contributions of distinct key populations to detritusphere carbon flow across microbial kingdoms. These objectives were addressed using a combination of PLFA- and rRNA-SIP as well as amplicon sequencing, providing an exceptional level of detail on the identities, succession and niche partitioning of key microbial populations in the detritusphere of an agricultural soil.

## Materials and Methods

### Soil

The soil originated from an agricultural field experiment located near Göttingen (Germany), which was designed to trace the flow of plant-derived carbon into the soil food web via C3 to C4 plant exchange (Kramer et al., 2012; Scharroba et al., 2012). A representative composite topsoil sample (0-10 cm) was taken from an area of ∼2 m^2^ within one control plot under wheat in October 2010. The C and N contents of the soil were 1.37 and 0.14%, respectively; soil pH_CaCl2_ was 6.0. Topsoil texture comprised 7% clay, 87% silt, and 6% sand. Further soil parameters can be found in Kramer et al. (2012). A homogeneous horizontal distribution of bacterial and fungal communities across replicate treatment plots of maize and wheat was previously shown for the site (Scharroba et al., 2012; Moll et al., 2015, 2016). Moreover, rapid and pronounced incorporation of plant-derived C into both bacterial and fungal biomass has been demonstrated for the soil (Kramer et al., 2012; Müller et al., 2016; Pausch et al., 2016).

### Microcosm Setup for SIP

Soil corresponding to 50 g dry weight was filled into steel cylinders (diameter = 5.5 cm, height = 4 cm). The four different substrates (glucose, cellulose, senescent maize leaves, and roots) were mixed into the soil and water content was adjusted to 60% water holding capacity. ^13^C treatments were set up with ^13^C-labeled substrates (>97 atom %; as determined by the supplier) as well as soil microcosms with unlabeled substrates (∼ natural abundance of ^13^C in maize plants; ^12^C treatments) and microcosms without substrate amendment (control). All substrates were purchased from IsoLife (Wageningen, Netherlands) and were derived from equivalent maize plants, with the exception of unlabeled glucose (Sigma-Aldrich, St. Louis, MO, USA). Amendments were added to the soil to a final amount of 12 mg C microcosm^-1^ (240 μg C g^-1^ soil), which represented only ∼2% of the intrinsic organic carbon (Kramer et al., 2012). To ensure a homogenous distribution of plant biomass amendments in the microcosms, materials were milled to <1 mm by the supplier. Cylinders were placed into air-tight glasses containing a small vessel attached to the lid to hold 1 M NaOH for absorbing evolved CO_2_. The microcosms were incubated in a climate chamber at 12°C, representing the long term autumn mean temperature at the site. Microcosms were sampled destructively after 2, 8, 16, and 32 days. For each time point, ^12^C treatments and controls were set up in triplicate, while ^13^C treatments were only set up with one microcosm per time point. See the SI for further details on the setup and substrate composition and also a scheme of the experimental setup and downstream workflow (Supplementary Figure S1).

### Inference of Substrate-Derived C-Pools

Carbon dioxide production, microbial biomass carbon (C_mic_), phospholipid fatty acids (PLFA) as well as the δ^13^C in CO_2_, C_mic_, and PLFAs were determined from ^12^C-microcosms as described in the SI. The following mixing model was used for calculation of the relative amounts of substrate-derived C in CO_2_, C_mic_, and PLFAs:

(1) $$\%\;\text{substrate-derived C}\;=\;((\delta_{\text{sample}}-\delta_{\text{reference}})/(\delta_{\text{substrate}}-\delta_{\text{soil}}))*100$$

δ_sample_ was the δ^13^C value of the respective sample and δ_reference_ was the mean δ^13^C value of controls (soil without substrate amendment). δ_substrate_ was the δ^13^C value of the amended material, and δ_soil_ the δ^13^C value of the C_org_ at the beginning of the experiment.

### RNA Extraction and rRNA Stable Isotope Probing (rRNA-SIP)

RNA was extracted from soil as described by Lueders et al. (2004) with minor modifications (see SI). RNA extracts of two time points were selected for SIP based on substrate mineralization and assimilation data. These were days 8 (high substrate use) and 32 (later stage of decomposition) for all treatments. Soil from triplicate ^12^C-microcosms was pooled and extracted as a composite sample for SIP. ^13^C-labeled rRNA was extracted from single microcosms per time point and treatment. Isopycnic centrifugation and gradient fractionation was done as previously described (Glaubitz et al., 2009; Kleindienst et al., 2014) with 750 ng of total RNA loaded into each gradient and collection of 12 RNA fractions after centrifugation.

### Fingerprinting and Sequencing of Density Resolved rRNA

Bacterial, fungal and protist rRNA in density-resolved SIP fractions (fractions 2 to 10 of all gradients) were first analyzed by T-RFLP fingerprinting following published protocols (Edel-Hermann et al., 2008; Euringer and Lueders, 2008; Pilloni et al., 2012). See the SI for full methodological detail. Based on rRNA fingerprinting results of gradient fractions, fractions 3 and 8 of all gradients were selected as representative for “heavy” and “light” rRNA and subjected to 454 amplicon sequencing (Pilloni et al., 2012; Dibbern et al., 2014; Kleindienst et al., 2014). All sequenced fractions were from a buoyant density of either ∼1.79 or ∼1.82 g ml^-1^ CsTFA, which are typical for light and heavy rRNA, respectively (Lueders et al., 2004). Processing and quality filtering of pyrosequencing reads was done as described in the SI. T-RFs for dominant taxa were predicted *in silico* via assembled contigs of amplicon reads as previously described (Pilloni et al., 2012). The sequencing of protist rRNA was restricted to gradients from one labile (glucose) and one complex (leaves) detritusphere substrate. All sequencing raw data have been deposited with the NCBI sequence read archive under SRA accession numbers SRP031455 (bacterial 16S rRNA reads), SRP031774 (protist 18S rRNA), and SRP033337 (fungal 18S).

### Calculation of Taxon-Specific Enrichment Factors in Labeled rRNA

To identify taxa involved in the assimilation of ^13^C from amended substrates within the different groups (bacteria, fungi, protists), sequencing read ‘enrichment factors’ (EF) in heavy rRNA fractions were inferred. EFs were calculated for all taxa with >2% read abundance in heavy rRNA fractions of at least one treatment and time point. This cutoff was chosen to cautiously constrain data interpretation to taxa with more notable rRNA enrichment and abundance in our experiment, and not to over-interpret EFs for taxa with low total read numbers. For protists only, all taxa were included in the calculation irrespective of their relative abundance in heavy fractions. The enrichment factors were calculated as follows:

(2) $$\text{EF}\;={\;}^{\text{13C}}\text{heavy}{/}^{\text{13C}}\text{light}{-}^{\text{12C}}\text{heavy}{/}^{\text{12C}}\text{light},$$

where ^13C^heavy and ^13C^light is the relative abundance of taxon-specific reads in heavy and light rRNA fractions of ^13^C treatments, and ^12C^heavy and ^12C^light is the same for respective ^12^C-controls. This approach is a variation of the ‘subtraction values’ of T-RFs in heavy vs. light rRNA used by Zumsteg et al. (2013), or of the ‘differential abundance’ approach for sequencing OTUs recently introduced for DNA-SIP (Pepe-Ranney et al., 2015). All taxa that showed an EF >0.5 in at least one treatment or time point were considered as ^13^C-labeled. For graphical display, the EFs were combined with relative read abundance of labeled taxa in heavy ^13^C-rRNA. Our EF-approach was supported by ‘classical’ labeling patterns as evident via abundance shifts of taxon-associated T-RFs across gradient fractions.

### Statistical Analysis

Cumulative CO_2_ production in ^12^C vs. ^13^C treatments were compared by one-way ANOVA. Substrate-derived C in CO_2_ over time was analyzed via ANOVA with date as repeated factor. A factorial ANOVA was done for comparison of relative incorporation of substrate-derived C into total microbial biomass as well as fungal and bacterial PLFAs (for each date separately). A post hoc test (Tukey HSD) was conducted for significance testing. Data were transformed if necessary to increase homogeneity of variance (tested by Levene‘s test). Similarity of biologically replicated PLFA community patterns for the different treatments and time points was assessed by multidimensional scaling (MDS) and discriminant function analysis (DFA; Marhan et al., 2007). All statistics were performed using STATISTICA 6.0 (Statsoft, Tulsa, OK, USA), details are described in the SI.

## Results

### Substrate Mineralization and ^13^C-Assimilation during Microcosm Incubation

Total turnover of ^13^C-labeled substrates as well as carbon flow into C_mic_ and CO_2_ depended on the complexity and recalcitrance of the added substrates. Approximately two-thirds of added glucose-C and cellulose-C were mineralized after 32 days, while only ∼45 and ∼12% of leaf- and root-C, respectively, were mineralized during the same time period (Supplementary Figure S2). Mineralization of ^12^C and ^13^C substrate amendments was not significantly different between microcosms (*F*_1,14_ = 0.004; *p* = 0.95).

**FIGURE 1:**
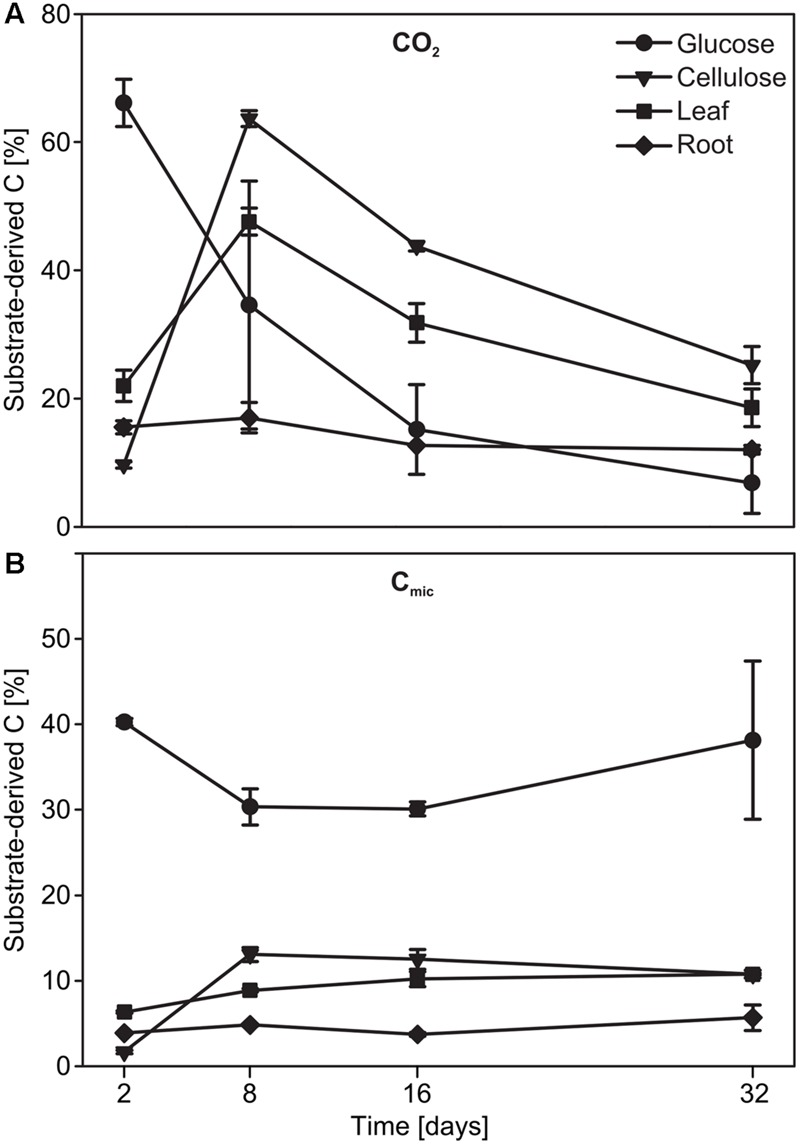
**Time course of the evolution of substrate-derived C in **(A)** CO_2_ and **(B)** C_mic_ of microcosms during incubation**.

Substrate mineralization and assimilation was quantified for replicated ^12^C treatments. This was possible via the ‘natural’ ^13^C-pulse of C4 plant material amended to a soil cultivated solely with C3 plants over the last decades (Kramer et al., 2012). On day 2, almost 70% of the CO_2_ produced originated from glucose in the respective treatments but was reduced to ∼7% by the end of the experiment (**Figure 1A**). In contrast, mineralization of cellulose and maize leaves peaked at day 8, with 64 and 48 %, respectively, of substrate-derived CO_2_. In the root treatment, the proportion of substrate-derived CO_2_ remained low (12-17 %) over time (**Figure 1A**; substrate effect: *F*_3,7_= 14.77, *p* = 0.002, date effect: *F*_3,21_= 71.49, *p* < 0.001). Resource-derived C in C_mic_ was highest for glucose (∼40%) after only 2 days of incubation (**Figure 1B**). Assimilation efficiency appeared much lower for the other substrates, and substrate-derived C was at a maximum of ∼15% for cellulose on day 8, and of ∼11 and ∼5% for leaf and root, respectively, at the end of the experiment (**Figure 1B**; substrate effect: *F*_3,32_= 236.54, *p* < 0.001, date effect: *F*_3,32_= 8.42, *p* < 0.001).

**FIGURE 2:**
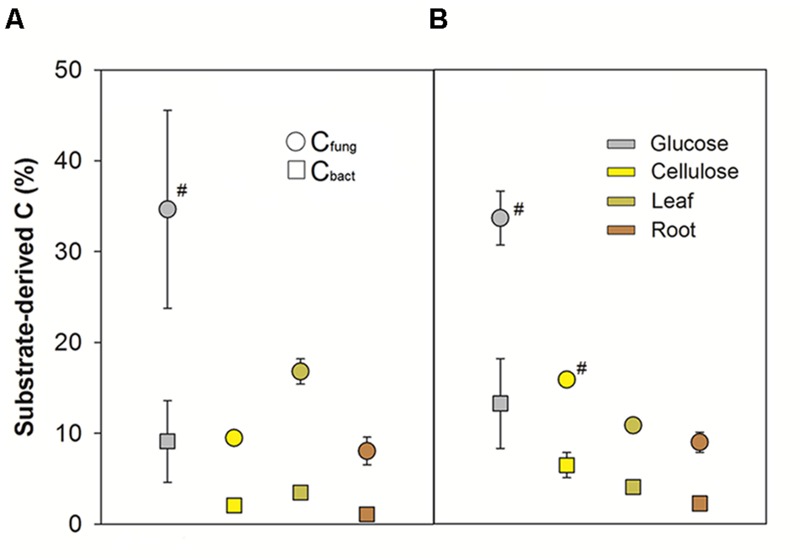
**Relative substrate-derived C in bacterial and fungal PLFAs after **(A)** 8 and **(B)** 32 days of incubation.** SE of measurements for triplicate microcosms per treatment and time point are indicated, except #: *n* = 2.

These distinctions in substrate-dependent carbon flow were also apparent in the allocation of substrate-derived C to bacteria and fungi as inferred via δ^13^C analyses of PLFAs in the ^12^C treatments. The fungal pool technically also included potential protist-derived fatty acids, as well as plant-derived fatty acids in the plant biomass treatments (Ruess and Chamberlain, 2010; Kaiser et al., 2015). Relative incorporation of substrate-derived C decreased with substrate complexity and recalcitrance (**Figure 2**, 8 days: *F*_3,15_= 4.79, *p* = 0.02; 32 days: *F*_3,14_= 24.41, *p* < 0.001). In general, a much higher incorporation into C_fung_ of substrate-derived C was observed (8 days: *F*_1,15_= 34.86, *p* < 0.001; 32 days: *F*_1,14_= 71.00, *p* < 0.001). Between the individual treatments, a slightly but significantly lower relative incorporation into C_bact_ vs. C_fung_ was observed for the root treatment after 32 days. However, pronounced quantitative distinctions in comparative kingdom-level substrate utilization were not observed for the labile vs. recalcitrant amendments (**Figure 2**).

Total PLFA community patterns were used to substantiate overall microbial community similarity between triplicate ^12^C- and singular ^13^C-microcosms per time point. Multivariate analyses of PLFA patterns showed a highly consistent overall community composition within replicate microcosms of all treatments and time points (Supplementary Figure S3). A significant separation (*p* < 0.001) was only observed between PLFA patterns of the day 8 glucose microcosms and all other samples (MDS axis 1). The secondary MDS axis discriminated earlier and later time points of incubation, but not treatments. More importantly, the grouping of all PLFA patterns from ^13^C-microcosms amongst the replicated ^12^C-microcosms (Supplementary Figure S3) indicated a comparable overall community composition between ^12^C- and ^13^C-amended microcosms.

### rRNA Stable Isotope Probing

**FIGURE 3:**
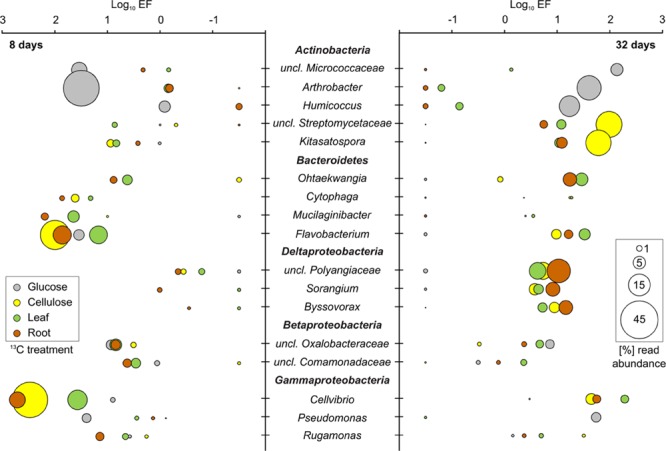
**^13^C-labeled bacterial taxa identified in SIP after 8 and 32 days of incubation.** Labeling was inferred via comparative sequencing read enrichment factors (EF) in heavy vs. light rRNA gradient fractions of ^13^C- and ^12^C-treatments. All bacterial taxa that showed an EF >0.5 in at least one treatment or time point were considered as ^13^C-labeled. Other taxa identified in sequencing libraries are not shown. EFs were combined with relative read abundance of labeled taxa in heavy ^13^C-rRNA. The EF of taxa with a negative read enrichment for given time points or treatments, but labeled in others, was manually set to a log_10_ of -1.5 for graphical display.

**FIGURE 4:**
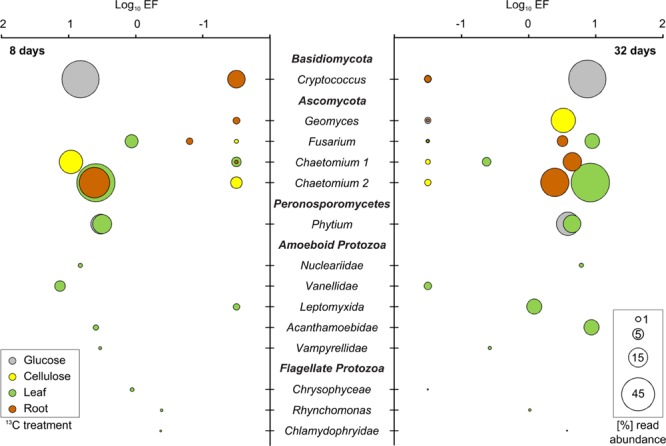
**^13^C-labeled fungal and protist taxa identified in SIP after 8 and 32 days of incubation.** All further details: see legend of **Figure 3**.

The incorporation of ^13^C from all substrates into bacterial and microeukaryote populations was confirmed by our initial screening of rRNA-SIP gradient fractions by RT-qPCR (SI) and by T-RFLP fingerprinting (Supplementary Figures S4–S6). For all substrates and time points, distinct T-RFs of bacterial, fungal, and even protist rRNA enriched in heavy vs. light fractions of ^13^C-gradients were observed. Such shifts were not observed in ^12^C-control gradients, a key criterion for substantiating ^13^C-labeling (Lueders et al., 2016). Subsequently, amplicon pyrosequencing of rRNA from representative heavy vs. light fractions was used to infer taxon-specific read EF for rRNA of labeled detritusphere microbes. We want to stress that the EFs as inferred here from non-replicated SIP gradients do not allow for a strict quantitative interpretation of taxon-specific buoyant density shifts as recently introduced for DNA-SIP (Hungate et al., 2015; Pepe-Ranney et al., 2016). Still, we are confident that they allow at least for a relative comparison of labeling efficiencies for distinct taxa between treatments and time points, and to identify the most important substrate consumers. Consistent with PLFA patterns (Supplementary Figure S3), fingerprinting (Supplementary Figures S4–S6) and sequencing analysis across all light rRNA fractions also supported a high similarity of total soil microbiota between treatments. EFs were also largely consistent for many labeled taxa between leaf and root treatments (**Figures 3** and **4**), which was not unexpected given the similar nature of these biomass amendments. To further support the inference of labeling via sequencing-based EFs, almost all taxa with a marked read enrichment could subsequently also be linked to respective T-RFs enriched in heavy ^13^C-rRNA (**Table 1**).

**Table 1:** Overview of ^13^C-labeled taxa identified in the detritusphere SIP experiment^∗^.

			Glucose		Cellulose		Leaf		Root	
		T-RFs	8 days	32 days	8 days	32 days	8 days	32 days	8 days	32 days
*Actinobacteria*	*Micrococcaceae*	*61, 71*	+	+						
	*Arthrobacter*	*61, 71, 159*	++	++			+		+	
	*Humicoccus*	*137, 145*	+	++						
	*Streptomycetaceae*	*69*				++	+	+		+
	*Kitasatospora*	*157*			+	++	+	+		+
*Bacteroidetes*	*Ohktaekwangia*	*205*					+	+	+	+
	*Cytophaga*				+		+		+	
	*Mucilaginibacter*	*524*					+		+	
	*Flavobacterium*	*79, 80, 84*	+		++	+	++	+	++	+
*Deltaproteobacteria*	*Myxobacteria*	*69, 500*				+		++		++
*Betaproteobacteria*	*Oxalobacteraceae*		+	+			+	+	+	
*Gammaproteobacteria*	*Cellvibrio*	*137, 486, 487*			++	+	++	+	++	+
	*Pseudomonas*	*490*	+	+						
	*Rugamonas*					+			+	
*Basidiomycota*	*Cryptococcus*	*560, 562, 564*	++	++						
*Ascomycota*	*Geomyces*	*768*				++				
	*Fusarium*						+	+		+
	*Chaetomium 1*	*708*			++					+
	*Chaetomium 2*	*708*					++	++	++	++
*Peronosporomycetes*	*Pythium*	*418, 421*	++	++			++	++		
*Protists*	*Vannellidae*	*429*					+			
	*Acanthamoebidae*	*484*					+	++		
	*Leptomyxida*							++		
	*Chlamydophryidae*	*412*					+	+		
	*Rhynchomonas*						+	+		

*^∗^++ Dominant labeling, enriched and abundant in heavy ^*13*^C-rRNA as evident via EFs and T-RFs; + ^*13*^C-labeled taxa with moderate enrichment or abundance in heavy RNA. Gray shading: gradients not analyzed for these lineages.*

### Labeled Bacterial rRNA

The different bacterial taxa incorporating ^13^C-label belonged mainly to three bacterial phyla: *Actinobacteria, Bacteroidetes* and *Proteobacteria* (**Figure 3**). Amongst the latter, mostly *Gammaproteobacteria* but also *Beta*- and *Deltaproteobacteria* were labeled. At day 8 of incubation, reads affiliated with *Arthrobacter* spp. were strongly enriched and abundant in heavy rRNA of the glucose treatment. However, unclassified *Micrococcaceae, Flavobacterium* spp., unclassified *Oxalobacteraceae* and *Pseudomonas* spp. were also enriched, albeit at lower read abundances. Interestingly, although glucose mineralization activity was mostly complete after 8 days (**Figure 1**), a dynamic labeling pattern was observed. After 32 days, reads related to *Flavobacterium* spp. were no longer enriched; while the EF of unclassified *Micrococcaceae, Humicoccus* and *Pseudomonas* spp. increased. However, *Arthrobacter* spp. remained the dominant taxon in heavy rRNA. *Cellvibrio*- and *Flavobacterium*-related reads were most highly enriched and abundant in heavy rRNA of the cellulose treatment after 8 days (**Figure 3**). While both were reduced after 32 days, sequences of unclassified *Streptomycetaceae* and *Kitasatospora* spp. became more important in labeled rRNA at this later time point. *Cytophaga* and *Rugamonas* spp. were identified as less enriched and/or abundant taxa after 8 and 32 days, respectively.

In leaf and root treatments, the highest enrichment was also observed for rRNA of *Flavobacterium* and *Cellvibrio* spp., but also for the less frequent *Mucilaginibacter* and *Cytophaga* spp. after 8 days. Strong enrichment was detected after 32 days for *Cellvibrio, Flavobacterium*, and *Ohtaekwangia* spp. in both leaf and root treatments. Interestingly, labeled rRNA of unclassified *Polyangiaceae* as well as of other *Myxobacteria* became noticeably more abundant and enriched within the leaf, root, and cellulose amendments after 32 days. Only one taxon (*Ohtaekwangia* spp., *Bacteroidetes*) appeared specifically enriched in the leaf and root treatments, especially after 32 days. Hardly any labeling was observed for this taxon under glucose or cellulose amendment. A summary of the most markedly labeled taxa identified in SIP is given in **Table 1**. Associated T-RFs, supportive of labeling for these lineages (Supplementary Figures S4–S6), are also listed.

### Labeled Fungal rRNA

Compared to bacteria, labeled fungi were less diverse and showed less pronounced enrichment in heavy rRNA. However, they showed more pronounced preferences for specific substrates. All fungal degraders of the added detritusphere substrates belonged to *Basidiomycota* and *Ascomycota* (**Figure 4**; **Table 1**). Glucose carbon was strongly assimilated by *Cryptococcus* spp. Surprisingly, other fungi were not labeled in this treatment. In turn, *Cryptococcus* yeasts were not labeled in any other treatment. Under cellulose amendment, enrichment was observed only for the *Chaetomium*-related phylotype 1 after 8 days, while *Geomyces* spp. were the only labeled fungi after 32 days. In the leaf and root treatments, a second *Chaetomium*-related phylotype was enriched and highly abundant after 8 days. Labeling was also observed for *Fusarium* spp. in both plant litter treatments after 32 days, as well as for the first *Chaetomium* phylotype in the root amendment. The labeling of associated T-RFs (**Table 1**) was also apparent in fingerprinting of density-resolved fungal rRNA (Supplementary Figure S6).

### Labeled Protist rRNA

Sequences of fungus-like protists within the *Peronosporomycetes* (*Pythium* spp.) were highly enriched in heavy rRNA in both the glucose and leaf treatments (**Figure 4**). Only these treatments were investigated for protists. Read abundance of protists other than *Pythium* was low in heavy rRNA of the glucose treatment and no further labeling was observed. In contrast, five amoeboid and three flagellate protozoan taxa appeared enriched in the leaf treatment (**Figure 4**). After 8 days, enrichment was found especially for the amoeboid *Vannellidae* (*Amoebozoa*) but also for the less abundant *Nucleariidae* (*Choanozoa*), *Acanthamoebidae* (*Amoebozoa*) and *Vampyrellidae* (*Cercozoa*). Labeling of *Vannellidae* was reduced after 32 days, while it was now more clearly detectable for *Acanthamoebidae* and *Leptomyxida* (*Amoebozoa*). Among the flagellate protozoa, members of the *Chrysophyceae* (*Bacillariophyceae*) and *Rhynchomonas* spp. (*Kinetoplastida*) also showed some rRNA enrichment, which was again also apparent for some associated T-RFs (**Table 1**; Supplementary Figure S6).

## Discussion

### Mineralization and Assimilation of Detritusphere Substrates

The diversity and succession of specific bacteria and microeukaryotes actively involved in the degradation of detritusphere substrates was investigated in a typical agricultural soil. Compared to several previous SIP studies that have focused on the degradation of cellulose and other substrate analogues in soil (Haichar et al., 2007; Schellenberger et al., 2010; Eichorst and Kuske, 2012; Štursová et al., 2012; Verastegui et al., 2014; Wang et al., 2015; Pepe-Ranney et al., 2016), the present work addressed distinct substrate complexities and their mineralization by detritusphere microbes up to the level of actual plant biomass. Additionally, detritusphere carbon flow and consumer succession was traced across microbial kingdoms. We show that overall mineralization was not significantly influenced by the isotopic composition of the amendments (Supplementary Figure S2), demonstrating that C utilization of the ^12^C- and ^13^C-substrate pairs was comparable over the experimental period. Compared to glucose (**Figure 1A**), the much less efficient mineralization and assimilation of C from plant biomass amendments (**Figures 1** and **2**; Supplementary Figure S2) appeared, as expected, directly related to the higher complexity of these materials (de Boer et al., 2005; Bertrand et al., 2006).

^13^C-PLFA analyses suggested that fungi incorporated more substrate-derived C than bacteria, consistent with the rapid and pronounced incorporation of plant-derived C into fungi already reported for the investigated soil (Kramer et al., 2012; Müller et al., 2016; Pausch et al., 2016), and also for other rhizosphere (Denef et al., 2007; Tavi et al., 2013) and detritusphere systems (Paterson et al., 2008; Alfredsson et al., 2016). The fungal 18:2ω6,9 PLFA biomarker (Ruess and Chamberlain, 2010) is also present in plants (Kaiser et al., 2015), which is why we must caution our quantitative interpretation especially for the leaf and root treatments after 8 days (**Figure 2**). However, we have evidence that plant-derived PLFA contributed only a minor fraction to the fungal pool from related studies with the same soil. Müller et al. (2016) followed the incorporation of ^13^C into fungal PLFA as well as ergosterol, a biomarker exclusive to fungal cell membranes. They reported a strict correlation of relative ^13^C incorporation into these independent fungal signature molecules. Moreover, our present study shows a higher relative incorporation of substrate-derived C into fungal PLFA also for the glucose and cellulose treatments, where plant-derived PLFA were not amended. Therefore, our data on ^13^C incorporation into bacterial and fungal PLFAs suggest that distinct bacterial and fungal energy channels were not apparent for labile vs. recalcitrant substrates, rejecting our first initial hypothesis for the investigated soil.

### Bacterial Key-Players in the Detritusphere

*Actinobacteria* have been previously shown to dominate sugar utilization for a number of soils (Schellenberger et al., 2010; Verastegui et al., 2014), thus our identification of *Arthrobacter* spp. and members of the *Micrococcaceae* as key glucose consumers was not surprising. *Pseudomonas* spp. are also well known to utilize glucose but more recalcitrant compounds as well, including lignin in soil (Goldfarb et al., 2011). A marked succession of bacterial glucose utilizers over time was not observed. This was in strong contrast to cellulose degradation, where an early cellulose degrading community dominated by *Cellvibrio* and *Flavobacterium* spp. profoundly shifted towards *Actinobacteria* after 32 days. While members of the *Bacteroidetes* and *Cellvibrio* spp. have been identified previously as cellulose consumers for a whole range of soils (Haichar et al., 2007; Schellenberger et al., 2010; Verastegui et al., 2014), such dynamic successions have not been observed (Pepe-Ranney et al., 2016). The secondary involvement of *Streptomycetes* in cellulose degradation seems plausible (Kämpfer, 2006), possible mechanisms of this succession will be discussed further down.

Most of the important cellulose decomposers were consistently apparent under plant biomass amendment, as well as their succession. This was not unexpected, given that cellulose was a major component of these amendments. After 32 days, unclassified *Polyangiaceae, Sorangium* and *Byssovorax* spp. (all *Deltaproteobacteria*) also emerged as labeled in the cellulose, leaf and root treatments. These *Myxobacteria* are known as saprotrophs but also as bacterial micropredators in soil (Johnke et al., 2014). Their strict secondary labeling could be indicative of feeding on labeled bacterial biomass (Lueders et al., 2006) of primary detritusphere components, which will also be discussed below.

### Detritusphere Fungi

In contrast to bacteria, detritusphere fungi showed more marked distinctions between glucose, cellulose and plant residue-utilizing taxa. *Cryptococcus* spp. dominated glucose utilization in our experiment, highlighting the role of these fast-growing yeasts as important competitors for labile carbon in the detritusphere. However, they remained unlabeled, surprisingly, in all other treatments. This is in contrast to Štursová et al. (2012), who reported *Cryptococcus* spp. as the main cellulolytic fungi in a forest soil. Either soil type or the use of bacterial vs. plant-derived cellulose amendments could potentially explain this distinction.

Early cellulose utilization was dominated by a *Chaetomium* phylotype and a marked succession to *Geomyces* spp. was observed after 32 days. Both genera have been previously identified by SIP as cellulose utilizers for a range of soils (Eichorst and Kuske, 2012; Štursová et al., 2012), but their succession has not been reported. A second *Chaetomium*-phylotype as well as *Fusarium* spp. were found as dominant fungi in plant residue degradation. The distinct substrate utilization patterns observed suggest that these fungi may harbor distinct exoenzymatic capabilities (Romani et al., 2006). *Cryptococcus* and *Chaetomium* spp. were also abundant in amplicon sequencing libraries generated directly from the investigated agricultural field (Moll et al., 2016), thus strengthening the link between labeling results obtained in laboratory microcosms and the detritusphere food web *in situ*.

### Labeled Protists

The flow of detritusphere carbon into protists was investigated only for one labile (glucose) and one complex (leaves) substrate (see SI). We are aware that this limits our interpretation of the effects of substrate complexity and recalcitrance on food web succession. However, due to the large overlaps of labeled bacteria detected in the non-glucose treatments (**Table 1**), we argue that this selection still makes it possible to infer the most fundamental distinctions in protist labeling. The only protists labeled in the glucose treatment were *Pythium* spp. These oomycetes are fungus-like protists within the *Stramenopiles* and known as important plant pathogens (Hendrix and Campbell, 1973), but can also act as pioneer saprotrophs on fresh plant residues in soil (Deacon, 1997). rRNA of *Pythium* spp. was labeled at both time points for maize leaf degradation also, indicating an important role of these protists in the detritusphere. Similarly, *Pythium* spp. has been found to be involved in carbon flow from rice root callus (Murase et al., 2012).

In contrast to the glucose treatment, labeling of diverse protozoa was observed in the leaf treatment. This labeling suggests a substantial flow of carbon to these higher trophic levels, despite the low C assimilation efficiency of protozoa and concomitant high respiration losses of ^13^CO_2_ (Crotty et al., 2012). ^13^C enrichment was mainly found in rRNA of amoeboid protozoa, again with marked succession over time. The amoeboid life style was likely more competitive in our soil microcosms, a distinction which seems warranted given the more marked labeling of flagellates reported for cellulose degradation in soil slurries (Chatzinotas et al., 2013). At least some of the labeled protozoa are known as fungivores, such as members of the *Leptomyxida* (Chakraborty and Old, 1982), the *Vampyrellidae* (Hess et al., 2012) and the *Chlamydophryidae* (Dumack et al., 2015). Although such fungivores are increasingly recognized as widespread in soils (Geisen et al., 2015), they are not yet embedded in food web concepts. Our study is the first to demonstrate a direct involvement of such fungivorous protozoa in detritusphere carbon flow.

### Substrate Complexity, Consumer Diversity, and Succession

The total number of dominantly labeled primary substrate consumers was low and not noticeably larger for any of the administered treatments (**Table 1**). Despite the fact that the investigated field site has been shown to host a horizontally homogeneous, but diverse soil microbiota (Scharroba et al., 2012; Dibbern et al., 2014; Moll et al., 2015; Moll et al., 2016), only a minority of taxa seemed to be involved in detritusphere decomposition. Thus in contrast to our second initial hypothesis, substrate complexity and recalcitrance were not directly correlated with consumer diversity. Although effects of substrate recalcitrance on overall microbial diversity have been reported (Nicolardot et al., 2007; Goldfarb et al., 2011), our results show that this does not necessarily apply to primary detritusphere consumers.

In contrary, we demonstrate that consumer succession and secondary trophic labeling increased with substrate complexity. Successional labeling was pronounced for most of the secondary consumers, which we believe discriminates them from primary consumers with low incorporation rates of labeled substrate. Marked successions of both bacterial and fungal key players were observed during cellulose decomposition and also for leaf and root treatments, albeit at decreasing kinetics (reflecting increasing substrate recalcitrance). Degradative succession has been intensively discussed for detritusphere fungi (Frankland, 1998) and recently also for bacteria (Pepe-Ranney et al., 2016) and is assumed to be primarily substrate-driven. In the present work, however, top-down controls may also have been involved in the observed successions. It is known that Gram-positive *Actinobacteria* are a non-preferred prey of protists due to their rigid cell walls and hyphal growth (Jousset, 2012). Potentially, the initial bursts of Gram-negative *Cellvibrio* and *Flavobacterium* populations in the cellulose and plant treatments may have been subject to intensive grazing by the diverse protozoa identified in the leaf treatment. Thus, niche space for more grazing-resistant actinobacterial cellulose consumers could have been generated. Consistently, the surprising lack of labeled protozoan rRNA in the glucose treatment may also reflect the dominant role of *Actinobacteria* in this amendment.

We also identified a prominent secondary succession of labeling within the *Myxobacteria*. These are known as saprotrophs but also as intra-bacterial micropredators in soil (Lueders et al., 2006; Johnke et al., 2014). Their consistent secondary labeling in the cellulose and plant biomass treatments does not allow to clearly differentiate both roles. Nevertheless, while intra-bacterial predation could indeed have occurred in the detritusphere, this question certainly merits further investigation.

### Bacterial and Fungal Energy Channels

Bacteria, fungi, and even protists were identified as primary consumers of all amendments, irrespective of substrate complexity or recalcitrance. This was suggested not only via rRNA-, but also PLFA-labeling, as also yeasts such as *Cryptococcus* (Marumo and Aoki, 1990) and protists such as *Pythium* (Erwin, 1973) harbor respective marker fatty acids. A preferential allocation of C from the labile amendments to bacteria was clearly not observed (**Figure 2**), thus rejecting the hypothesis of distinct bacterial and fungal energy channels for the investigated soil. Substrate partitioning may vary for different soils, as both a faster and more pronounced incorporation of C from labile and soluble amendments into bacterial PLFAs (Paterson et al., 2008), and also of labile root exudates into fungal PLFAs (Butler et al., 2003; Denef et al., 2007) have been reported. For the agricultural soil investigated here, our results promote the perspective of simultaneous and overlapping substrate utilization by bacteria, fungi, and protists in the detritusphere, irrespective of resource quality.

## Conclusion

Substrate complexity and recalcitrance were identified as primary drivers of consumer succession and secondary trophic labeling over time. If the detritusphere is envisioned as a continuum of substrate inputs and degradation in discrete niches, such successional effects will essentially increase consumer diversity for more recalcitrant substrates. However, the mechanisms are distinct from a direct substrate-driven selection of primary consumers and may be partly under top-down control. Our results suggest a role of protists not only as bacterivores, but also as fungivores and even primary saprotrophs in the investigated detritusphere food web. Likewise, *Myxobacteria* may also have been active as bacterivores. Such intra- and trans-kingdom feedbacks and successions await better incorporation into conceptual models of soil food webs. Here, the direct linking of key microbial populations to relevant decomposition processes is a major challenge (Trivedi et al., 2013) which can be addressed using SIP, as shown here. Trophic interactions and succession rather than substrate-defined energy channels may be a vital asset to existing ecosystem models (Moore et al., 2005; McGuire and Treseder, 2010), possibly facilitating more accurate predictions of soil carbon cycling in the future.

## Author Contributions

EK, TL, and SM designed the experiments. SK, DD, JM, and MH conducted the experiments. SK, DD, JM, MH, RK, DK, SM, TU, and TW analyzed data. SK, EK, MB, FB, and TL wrote the manuscript with contributions from all authors.

## Conflict of Interest Statement

The authors declare that the research was conducted in the absence of any commercial or financial relationships that could be construed as a potential conflict of interest.
