# Supplementary material for: Resource Partitioning between Bacteria, Fungi, and Protists in the Detritusphere of an Agricultural Soil
Source: Front Microbiol. 2016 Sep 26;7:1524. doi: 10.3389/fmicb.2016.01524 (PMC5035733; doi:10.3389/fmicb.2016.01524)
Supplement: Supplementary file 1 [file Presentation_1.PDF]

Resource partitioning between bacteria, fungi and protists in the detritusphere of an agricultural soil

Susanne Kramer, Dörte Dibbern, Julia Moll, Maike Huenninghaus,

Robert Koller, Dirk Krueger, Sven Marhan, Tim Urich, Tesfaye Wubet,

Michael Bonkowski, François Buscot, Tillmann Lueders, Ellen Kandeler

Correspondence: Tillmann Lueders, Institute of Groundwater Ecology, Helmholtz Zentrum München - German Research Center for Environmental Health, Ingolstädter Landstr. 1, D-85764 Neuherberg. E-mail: tillmann.lueders@helmholtz-muenchen.de.

Supporting Information (SI)

Supplementary Materials and Methods 2

Supplementary References 11

Supplementary Tables 14

Supplementary Figures 15

Supplementary Materials and Methods

Microcosm experiment

The soil was sieved (< 2 mm), stored at 4 °C for a few days and water holding capacity was determined. Two weeks before the start of the SIP experiment, soil was pre-incubated at the experimental temperature of 12 °C. Soil bulk density was set at 1.4 g cm^{-3}. During incubation, microcosms were weighed repeatedly to control water content. No significant decrease in soil water content was observed, adjustment was not necessary. Complex ^{12}C- and ^{13}C-substrates were purchased from IsoLife (Wageningen, Netherlands) and were derived from equivalent maize plants. Maize leaves and roots were obtained from 12 week old plants (variety "Yukon Chief") grown by IsoLife. The amended plant material had a C/N ratio of 82 (^{13}C-leaves), 54 (^{12}C-leaves), 50 (^{13}C-roots) and 37 (^{12}C-roots). ^{13}C-cellulose (58% glucose, 4.4% residual lignin, 2,100 DP, 340 kD average MW) and ^{12}C-cellulose (59% glucose, 4.2% residual lignin, 2,200 DP, 356 kD average MW) were also maize-derived and purchased from IsoLife. ^{12}C treatments and controls were run in triplicate for each time point, while ^{13}C treatments were only set up with one microcosm per time point. We are aware of this limitation, but full biological replication of ^{13}C microcosms was not possible for budget reasons. Still, the generally high similarity of microbial communities between the triplicate ^{12}C and singular ^{13}C microcosms for each treatment and time point (Fig. S2), same as between light rRNA fractions for all parallel SIP gradients (Figs. S3 – S5), as well as the replicate ^{13}C-microcosms subjected to successive destructive sampling allow for a reasonable level of confidence in the interpretation of results, to our opinion. After incubation, microcosms were destructively sampled, the soil was homogenized and subsamples were stored at -80 °C for RNA extraction, and at -24 °C for all other analyses.

CO_2 production and microbial biomass C

CO_2 production was determined by titration over the entire incubation time in increasing time distances between measurements (Marhan et al., 2008). CO_2 was trapped in 1 M NaOH and precipitated with 0.5 M $BaCl_2$. The remaining NaOH was titrated with 0.1 M HCl with phenolphthalein indicator (Marhan et al., 2008). After sampling for titration, lids of the microcosms were left open to allow gas exchange, thus the measurement of CO_2 production was non-accumulative over time intervals. Another part of the precipitated $BaCO_3$ was used for $\delta^{13}C$ determination of the evolved CO_2 (only in the ^{12}C treatments).

Microbial biomass C (C_{mic}) was determined by chloroform-fumigation extraction (Vance et al., 1987). 3 g (fresh weight) of homogenized soil was extracted with 0.025 M K_2SO_4 [1:4 soil solution ratio (w/v)], shaken for 30 min at 250 rev min^{-1} on a horizontal shaker and centrifuged for 30 min at 4,422 x g. Parallel subsamples were fumigated with ethanol-free chloroform in a desiccator for 24 h before extraction. Organic C in the supernatants was measured with a DOC / TN-analyser (Dimatoc 100, Dimatec, Essen, Germany).

Phospholipid fatty acid analysis

Phospholipid fatty acid (PLFA) analysis was conducted to infer overall similarity of microbial communities across replicate microcosms, time points and treatments, as well as to quantify relative amounts of substrate-derived C assimilated by bacteria vs. fungi (C_{bact} & C_{fung}). PLFAs were extracted from triplicate ^{12}C microcosms as well as the ^{13}C microcosm per treatment at days 8 and 32 using the procedure described by (Frostegård et al., 1993). Per microcosm, lipids of 6 g soil were extracted with a Blight & Dyer solution (chloroform, methanol, citrate buffer (pH 4); 1:2:0.8; v/v/v) and further separated into glycol-, neutral lipids and phospholipid fatty acids using silica acid SPE cartridges (Bond Elut SI, 500 mg, 3 ml, Agilent Technologies Inc., Santa Clara, USA). Methanolysis of PLFAs was done with 0.2

M methanolic KOH (Ruess et al., 2007). The fatty acid methyl esters (FAMEs) were dissolved in isooctane and measured in an AutoSystem XL gas chromatograph (Perkin-Elmer Corporation, Norwalk, USA) equipped with a HP-5 capillary column (cross-linked 5 % phenyl methyl siloxane; 50 m x 0.2 mm, film thickness of 0.33 µm) and a flame ionization detector. The temperature program started with 70 °C for 2 min, increased with 30 °C min^{-1} to 160 °C, further 3 °C min^{-1} to 280 °C and held for 15 min. The injection temperature was 260 °C. Helium was used as carrier gas. The following PLFAs were considered to be mostly of microbial origin in our detritusphere soil: 14:0, i15:0, a15:0, 15:0, i16:0, 16:1ω7, 16:1ω6, 16:1ω5, 16:0, i17:0, cy17:0, 17:0, 18:2ω6, 18:2ω6t/18:1ω9/18:3ω3, 18:1ω7/18:1ω9t, 18:0, cy19:0, 20:4ω6 (Frostegård et al., 1993; Zelles, 1999) and were used to infer microbial community structure.

$\delta^{13}C$ determination of CO_2, C_{mic} and PLFAs

For $\delta^{13}C$ determination in evolved CO_2, precipitated $BaCO_3$ was washed with 10 ml deionised H_2O and centrifuged at 250 x g, after which the supernatant was discarded. This was repeated three times until all residual NaOH was removed. Pellets of $BaCO_3$ were then dried at 60 °C for two days and 0.3 – 0.6 mg was weighed into tin capsules. For analysis of $\delta^{13}C$ in C_{mic} (only in ^{12}C treatments) 10 ml aliquots of the supernatants of both non-fumigated and fumigated samples were dried in a rotary vacuum evaporator (RVC 2-25, Martin Christ, Osterode am Harz, Germany) at 60 °C (Marhan et al., 2010). The remnant was ground and weighed into tin capsules within a range of 7 – 30 mg (minimum of 10 µg C per capsule).

$\delta^{13}C$ measurements of CO_2 and C_{mic} were done with an elemental analyzer (Euro EA 3000, EuroVector, Milan, Italy) coupled with an isotope ratio mass spectrometer (IRMS, Delta Plus XP, Thermo Finnigan MAT, Bremen, Germany). Glutamic acid USGS-40 (IAEA, Vienna; $\delta^{13}C$ -26.39 ± 0.04 ‰) was used as reference material for calibration of CO_2 reference gas. Acetanilide (C_8H_9NO, Merck, Darmstadt) was used as a secondary laboratory

reference material for internal calibration. $\delta^{13}C$ values are expressed relative to Vienna Pee Dee belemnite (V-PDB). The following equation was used for the calculation of the $\delta^{13}C$ of C_{mic}:

$$\delta^{13}C_{mic} = (c_{nf} \times \delta_{nf} - c_f \times \delta_f) / (c_{nf} - c_f),$$

whereas c_{nf} and c_f were the corresponding extracted organic C contents (µg C g^{-1}soil) of the non-fumigated and the fumigated sample, and δ_{nf} and δ_f are the corresponding $\delta^{13}C$ values.

For determination of $\delta^{13}C$, the PLFA samples were fractionated with Ag^+-SPE cartridges (6 ml, Supelco, Palo Alto, USA) according to (Kramer et al., 2008). Before use, cartridges were conditioned with acetone and n-hexane. A stepwise elution with 6 ml n-hexane containing increasing amounts of acetone (99:1, v/v; 96:4, v/v; 90:10, v/v and 0:100, v/v) separated FAMEs into four different fractions: The first fraction contained saturated FAMEs, the second and third fraction monoenoic trans and cis FAMEs and the forth fraction dienoic FAMEs. The first and fourth fraction was kept and evaporated to dryness by means of N_2 and re-dissolved in 70 µl isooctane. A gas chromatograph (6890 series, Agilent Technologies, Santa Clara, USA) equipped with a VS-23MS capillary column (Varian Medical Systems, Palo Alto, USA; 30 m x 250 µm, film thickness of 0.25 µm) was coupled with a gas chromatography-combustion III Interphase (Thermo Finnigan, Waltman, USA) to a Delta Plus XP mass spectrometer (Thermo Finnigan MAT, Bremen, Germany) to determine the $\delta^{13}C$ values of the FAMEs. The oven temperature was 290 °C and the injector temperature 250 °C. The temperature program started with 80 °C for 2 min, increased with 10 °C min^{-1} to 140 °C, further increased with 4 °C min^{-1} to 240 °C and held for 5 min. The $\delta^{13}C$ values of all FAMEs were corrected for the addition of a methyl group by using a mass balance equation (Denef et al., 2007). The $\delta^{13}C$ value of methanol used for methylation was -40.23 ‰. The fatty acids i15:0, a15:0, i16:0, i17:0 and cy17:0 were used as specific biomarkers for bacteria, whilst 18:2ω6,9c was used for fungi (Ruess and Chamberlain, 2010).

RNA extraction

Total nucleic acids were extracted from the soil following a previously described procedure (Lueders et al., 2004) with minor modifications: 0.4 g (fresh weight) of soil were used and bead beating was done in the presence of sodium phosphate, sodium dodecyl sulphate and phenol-chloroform-isoamyl alcohol (25:24:1, pH 8). All centrifugation steps were conducted at 20,000 x g and 4 °C for 5 min. Extracted total NAs were dissolved in 80 µl EB buffer (Qiagen GmbH, Hilden, Germany). Silica gel columns (DyeEx 2.0 Spin Kit; Qiagen) were used for further purification and elimination of humics. DNA was removed by digestion with DNAse I (Promega, Madison, WI, USA) following manufacturer protocols. Afterwards, RNA was precipitated with 2 vol. PEG solution (30% (w/v) polyethylene glycol 6000, 1.6 mM NaCl) and centrifugation for 30 min at 4 °C and 20,000 x g. RNA pellets were washed once with ice cold 70% (v/v) ethanol, air-dried and dissolved in 50 µl EB Buffer. The resulting RNA was quantified using the RiboGreen quantification kit (Life Technologies, Carlsbad, CA).

Quantitative analysis of SIP gradient fractions

After fractionation and precipitation of density-resolved rRNA, bacterial rRNA was quantified in gradient fractions via RT-qPCR as described in (Glaubitz et al., 2009). The initial screening revealed a ^{13}C-dependent increase of bacterial rRNA in "heavy" fractions (data not shown). The buoyant density of the bulk rRNA peak remained unchanged in 'light' fractions (~1.78 – 1.79 g/ml CsTFA), indicating that only specific subsets of soil microbiota were actively involved in the utilization of the labeled detritusphere substrates.

Terminal restriction fragment length polymorphism (T-RFLP) fingerprinting

Bacterial, fungal and protist rRNA populations in resolved SIP fractions were analyzed by T-RFLP fingerprinting. See Table S1 for a summary of all utilized PCR assays.

Bacterial communities were analyzed with primers Ba27f-FAM / 907r and subsequent *Msp*I digestion as previously described (Pilloni et al., 2011). Protistan communities were characterized with primers Euk20f-FAM / Euk519r and *Bsh*1236I digestion, in a minor modification of the assay originally published by (Euringer and Lueders, 2008). Reverse transcription of eukaryotic rRNA and PCR amplification was done with the Brilliant III Ultra-Fast SYBR Green one-step RT-qPCR Master Mix (Agilent Technologies Inc., Santa Clara, California) as specified by the manufacturer with 0.3 µl of each primer and 2 µl of RNA template. For both bacterial and protistan amplicons, digests were purified and separated by capillary electrophoresis (Pilloni et al., 2011).

Fungal communities were characterized with primers nu-SSU-0817-5'- FAM / nu-SSU-1536-3' (Borneman and Hartin, 2000) and *Msp*I digestion after (Edel-Hermann et al., 2008). First-strand cDNA was prepared using the RevertAid Premium First Strand cDNA Synthesis kit and provided random hexamer primers (Thermo Scientific, St. Leon-Rot, Germany). PCR was performed in 40 µl reaction mixtures containing 20 µl 2x GoTaq Green Master mix (Promega, Madison, WI, USA), 20 µM of each primer and 3 µl template cDNA. PCR products were purified using 5Prime PCRExtract Mini Kit (5PRIME, Inc., Bucksfield Road, Gaithersburg, USA) followed by *Msp*I (Thermo Scientific, St. Leon-Rot, Germany) digestion of 80 ng PCR product for two hours according to the manufacturer's protocol. After purification of the reaction mixture by ethanol precipitation, fungal communities were analyzed on a ABI 3730xl capillary electrophoresis sequencer (Applied Biosystems, Foster City, CA, USA) as described previously (Scharroba et al., 2012).

Bacterial and protistan raw T-RFLP data were further processed with the T-REX online software (Culman et al., 2009). Background noise filtering (Abdo et al., 2006) was on default factor 1 for peak heights and the clustering threshold for aligning peaks across the samples was set to 1 using the default alignment method of T-Align (Smith et al., 2005). Relative T-RF abundance was inferred from peak heights. For reduction of data complexity,

T-RFs that occurred in less than 5% of the samples were excluded from further analysis. Fungal T-RFLP data with all peaks above a threshold of 100 fluorescence units were binned and normalized with an automatic binning script (Ramette, 2009) using R version 2.12.2 (R Development CoreTeam, 2012). The binning frame with highest correlation values between samples and a window size of two was chosen. Peaks with a relative abundance below 0.1% were discarded as background noise.

Amplicon pyrosequencing

Tagged bacterial rRNA amplicons for sequencing were generated as reported previously (Pilloni et al., 2012; Dibbern et al., 2014; Kleindienst et al., 2014), adapting the workflow to rRNA templates instead of DNA. Shortly, RT-PCR was done with the same primers under identical conditions as for fingerprinting, but applying amplicon fusion primers with respective primer A or B adapters, key sequence and multiplex identifiers (MID) as reported (Pilloni et al., 2012). Amplicons were purified and pooled in equimolar 10^9 μl^{-1} concentration, and emulsion PCR, emulsion breaking and sequencing were performed as previously described in detail (Pilloni et al., 2012) following manufacturer protocols using a 454 GS FLX pyrosequencer using Titanium chemistry (Roche, Penzberg, Germany). Bidirectional reads were quality-trimmed and filtered as previously described (Pilloni et al., 2012), and reads shorter than 250 bp after trimming were excluded from further analysis. Classification of bacterial taxa was done with the RDP classifier (Wang et al., 2007).

Protistan sequencing amplicons were only generated for the glucose and leaf treatments, same as for fingerprints. Unfortunately, fractionated gradient RNA from the cellulose and root treatments was lost during transport between different labs, and could no longer be subjected to protistan rRNA analyses. We are aware that this limits our interpretation of the effects of substrate complexity and recalcitrance on food web succession. However, due to the large overlaps of labeled bacteria detected in the non-glucose treatments

(Table 1), we argue that this selection still allows inferring fundamental distinctions in protist labeling. Amplicon preparation for protists was done as for bacteria but with modified PCR conditions (Table S1) and with the same Brilliant III Ultra-Fast RT-qPCR Master Mix (Agilent Technologies, Santa Clara, USA) and eukaryote primers (now as fusion primers) as used for respective T-RFLP fingerprinting. Quality-trimming and filtering was the same as for bacterial reads. 18S rRNA amplicon sequences were taxonomically analyzed with the CREST toolbox (Lanzén et al., 2012). In brief, the amplicons were taxonomically assigned by MEGAN analysis of BLASTN files against the SilvaMod SSU rRNA reference database (LCA parameters: min. bit score 330, min. support 1, top percent 2; 50 best blast hits).

For sequencing of fungal rRNA, tagged amplicons were amplified as described for the fungal T-RFLP analyses, except of using the unlabeled forward primer nu-SSU-0817-5' combined with the fusion primer B (modified after (Becklin et al., 2012). MID barcodes were inserted between the A primer and primer nu-SSU-1536-3' to allow post-sequencing sample identification. PCR products were purified from agarose gels using the QIAquick Gel Extraction kit (Qiagen, Valencia, CA, USA). The clean amplicons were quantified using the Quant-IT PicoGreen dsDNA Reagent kit (Life Technologies GmbH, Darmstadt, Germany), diluted to 10^9 molecules /µl and equimolarly pooled into an amplicon library following manufacturer protocols (Roche). The fungal libraries were sequenced in one 1/4th plate using GS-FLX+ sequencer (Roche). The pyrosequences were processed and quality filtered using mothur (Schloss et al., 2009). Barcodes and primers were trimmed and sequences were extracted based on 100% barcode similarity, an average quality score of 20, read length of 300 bp after trimming of the last 30 bp and homo-polymers of 8 bases. The chimera check command 'uchime' with 'template self' was used to detect potentially chimeric sequences and remove them from the dataset. Sequences were then clustered to operational taxonomic units (OTUs) using 'cd-hit-est' with a threshold of 97 % pairwise identity. Representative sequences in the respective clusters were extracted and taxonomically assigned according to

the arb silva eukaryotic taxonomy using the 'classify seqs' command of mothur with 80 % cutoff. The taxonomic position of the dominant fungal OTUs was manually verified using the NCBI blast database. Fungal in-vitro T-RFs were generated based on full length clone library sequences done for heavy fraction 3 with the software TRiFLe (Junier et al., 2008).

For the linking of T-RF and pyrosequencing data, matching sequences from bidirectional amplicon pools were assembled into contigs with the SEQMAN II software (DNAStar) using assembly thresholds of at least 97 % sequence similarity over a 50 bp match window for T-RF prediction (Pilloni et al., 2012). Only contigs containing at least one forward and one reverse read were used to predict *in-silico*-T-RFs for dominating consensus phylotypes using TRiFLe (Junier et al., 2008).

Statistical Analysis

Similarity of biologically replicated PLFA community patterns for the different treatments and time points was assessed by multidimensional scaling (MDS) and discriminant function analysis (DFA) (Marhan et al., 2007). In brief, a square matrix of nonparametric Gamma correlation (analogous to Kendall τ) was calculated from the relative abundances of all PLFAs. This matrix was analyzed by multidimensional scaling, i.e. an ordination technique which rearranges objects in a maximally nine-dimensional space, so as to arrive at a configuration that best approximates the observed distances. The number of meaningful dimensions was evaluated by comparing actual stress values, representing a measure for the loss of information when the data are fitted into the n-dimensional space, with the theoretical exponential function of stress. The coordinates of the samples in the n-dimensional space were used for DFA, with treatment as a grouping variable. Squared Mahalanobis distances between group centroids and reliability of sample classification were determined. Two significant discriminatory roots were derived (MDS with 9 dimensions) and the results of

DFA were graphically presented in two dimensions (axis 1 and 2). Statistical analyses were done with the software STATISTICA 6.0 (Statsoft, Tulsa, OK, USA).

Supplementary References

Abdo, Z., Schuette, U.M.E., Bent, S.J., Williams, C.J., Forney, L.J., and Joyce, P. (2006). Statistical methods for characterizing diversity of microbial communities by analysis of terminal restriction fragment length polymorphisms of 16S rRNA genes. *Environ. Microbiol.* 8**,** 929-938. doi: doi:10.1111/j.1462-2920.2005.00959.x.

Becklin, K.M., Hertweck, K.L., and Jumpponen, A. (2012). Host identity impacts rhizosphere fungal communities associated with three alpine plant species. *Microbial Ecol.* 63**,** 682-693. doi: 10.1007/s00248-011-9968-7.

Borneman, J., and Hartin, R.J. (2000). PCR primers that amplify fungal rRNA genes from environmental samples. *Appl. Environ. Microbiol.* 66**,** 4356-4360. doi: 10.1128/AEM.66.10.4356-4360.2000

Culman, S.W., Bukowski, R., Gauch, H.G., Cadillo-Quiroz, H., and Buckley, D.H. (2009). T-REX: software for the processing and analysis of T-RFLP data. *BMC Bioinformatics* 10**,** 171-171. doi: 10.1186/1471-2105-10-171.

Denef, K., Bubenheim, H., Lenhart, K., Vermeulen, J., Cleemput, O.V., Boeckx, P., et al. (2007). Community shifts and carbon translocation within metabolically-active rhizosphere microorganisms in grasslands under elevated CO_2. *Biogeosci.* 4**,** 769-779. doi: 10.5194/bg-4-769-2007.

Dibbern, D., Schmalwasser, A., Lueders, T., and Totsche, K.U. (2014). Selective transport of plant root-associated bacterial populations in agricultural soils upon snowmelt. *Soil Biol. Biochem.* 69**,** 187-196. doi: 10.1016/j.soilbio.2013.10.040.

Edel-Hermann, V., Gautheron, N., Alabouvette, C., and Steinberg, C. (2008). Fingerprinting methods to approach multitrophic interactions among microflora and microfauna communities in soil. *Biol. Fertil. Soils* 44**,** 975-984. doi: 10.1007/s00374-008-0287-1

Euringer, K., and Lueders, T. (2008). An optimised PCR/T-RFLP fingerprinting approach for the investigation of protistan communities in groundwater environments. *J Microbiol. Meth.* 75**,** 262-268. doi: 10.1016/j.mimet.2008.06.012.

Frostegård, Å., Bååth, E., and Tunlio, A. (1993). Shifts in the structure of soil microbial communities in limed forests as revealed by phospholipid fatty acid analysis. *Soil Biol. Biochem.* 25**,** 723-730. doi: 10.1016/0038-0717(93)90113-P.

Glaubitz, S., Lueders, T., Abraham, W.-R., Jost, G., Jurgens, K., and Labrenz, M. (2009). ^{13}C-isotope analyses reveal that chemolithoautotrophic *Gamma*- and *Epsilonproteobacteria* feed a microbial food web in a pelagic redoxcline of the central Baltic Sea. *Environ. Microbiol.* 11**,** 326-337. doi: 10.1111/j.1462-2920.2008.01770.x.

Junier, P., Junier, T., and Witzel, K.-P. (2008). TRiFLe, a program for *in silico* terminal restriction fragment length polymorphism analysis with user-defined sequence sets. *Appl. Environ. Microbiol.* 74**,** 6452-6456. doi: 10.1128/aem.01394-08.

Kleindienst, S., Herbst, F.-A., Stagars, M., von Netzer, F., von Bergen, M., Seifert, J., et al. (2014). Diverse sulfate-reducing bacteria of the *Desulfosarcina/Desulfococcus* clade are the key alkane degraders at marine seeps. *ISME J* 8**,** 2029-2044. doi: 10.1038/ismej.2014.51.

Kramer, J.G., Hernandez, M., Cruz-Hernandez, C., Kraft, J., and Dugan, M.R. (2008). Combining results of two GC separations partly achieves determination of all *cis* and *trans* 16:1, 18:1, 18:2 and 18:3 except CLA isomers of milk fat as demonstrated using Ag-ion SPE fractionation. *Lipids* 43**,** 259-273. doi: 10.1007/s11745-007-3143-4.

Lanzén, A., Jørgensen, S.L., Huson, D.H., Gorfer, M., Grindhaug, S.H., Jonassen, I., et al. (2012). CREST – Classification resources for environmental sequence tags. *PLoS ONE* 7**,** e49334. doi: 10.1371/journal.pone.0049334.

Lueders, T., Manefield, M., and Friedrich, M.W. (2004). Enhanced sensitivity of DNA- and rRNA-based stable isotope probing by fractionation and quantitative analysis of isopycnic centrifugation gradients. *Environ. Microbiol.* 6**,** 73-78. doi: 10.1046/j.1462-2920.2003.00536.x.

Marhan, S., Demin, D., Erbs, M., Kuzyakov, Y., Fangmeier, A., and Kandeler, E. (2008). Soil organic matter mineralization and residue decomposition of spring wheat grown under elevated CO2 atmosphere. *Agr.Ecosyst.Environ.* 123**,** 63-68. doi: 10.1016/j.agee.2007.04.001.

Marhan, S., Kandeler, E., Rein, S., Fangmeier, A., and Niklaus, P.A. (2010). Indirect effects of soil moisture reverse soil C sequestration responses of a spring wheat agroecosystem to elevated CO2. *Glob.Change Biol.* 16**,** 469-483. doi: 10.1111/j.1365-2486.2009.01949.x.

Marhan, S., Kandeler, E., and Scheu, S. (2007). Phospholipid fatty acid profiles and xylanase activity in particle size fractions of forest soil and casts of *Lumbricus terrestris* L. (*Oligochaeta, Lumbricidae*). *Appl. Soil Ecol.* 35**,** 412-422. doi: 10.1016/j.apsoil.2006.06.003.

Pilloni, G., Granitsiotis, M.S., Engel, M., and Lueders, T. (2012). Testing the limits of 454 pyrotag sequencing: reproducibility, quantitative assessment and comparison to T-RFLP fingerprinting of aquifer microbes. *PLoS ONE* 7**,** e40467. doi: 10.1371/journal.pone.0040467.

Pilloni, G., von Netzer, F., Engel, M., and Lueders, T. (2011). Electron acceptor-dependent identification of key anaerobic toluene degraders at a tar-oil-contaminated aquifer by Pyro-SIP. *FEMS Microbiol. Ecol.* 78**,** 165-175. doi: 10.1111/j.1574-6941.2011.01083.x.

R Development CoreTeam (2012). "R: A language and environment for statistical computing. Vienna: R foundation for statistical computing ".).

Ramette, A. (2009). Quantitative community fingerprinting methods for estimating the abundance of operational taxonomic units in natural microbial communities. *Appl. Environ. Microbiol.* 75**,** 2495-2505. doi: 10.1128/aem.02409-08.

Ruess, L., and Chamberlain, P.M. (2010). The fat that matters: Soil food web analysis using fatty acids and their carbon stable isotope signature. *Soil Biol. Biochem.* 42**,** 1898-1910. doi: 10.1016/j.soilbio.2010.07.020.

Ruess, L., Schütz, K., Migge-Kleian, S., Häggblom, M.M., Kandeler, E., and Scheu, S. (2007). Lipid composition of Collembola and their food resources in deciduous forest stands—Implications for feeding strategies. *Soil Biol. Biochem.* 39**,** 1990-2000. doi: 10.1016/j.soilbio.2007.03.002.

Scharroba, A., Dibbern, D., Hünninghaus, M., Kramer, S., Moll, J., Butenschoen, O., et al. (2012). Effects of resource availability and quality on the structure of the micro-food web of an arable soil across depth. *Soil Biol. Biochem.* 50**,** 1-11. doi: 10.1016/j.soilbio.2012.03.002.

Schloss, P.D., Westcott, S.L., Ryabin, T., Hall, J.R., Hartmann, M., Hollister, E.B., et al. (2009). Introducing mothur: open-source, platform-independent, community-supported software for describing and comparing microbial communities. *Appl. Environ. Microbiol.* 75**,** 7537-7541. doi: 10.1128/aem.01541-09.

Smith, C.J., Danilowicz, B.S., Clear, A.K., Costello, F.J., Wilson, B., and Meijer, W.G. (2005). T-Align, a web-based tool for comparison of multiple terminal restriction fragment length polymorphism profiles. *FEMS Microbiol. Ecol.* 54**,** 375-380. doi: 10.1016/j.femsec.2005.05.002.

Vance, E.D., Brookes, P.C., and Jenkinson, D.S. (1987). An extraction method for measuring soil microbial biomass C. *Soil Biol. Biochem.* 19**,** 703-707. doi: 10.1016/0038-0717(87)90052-6.

Wang, Q., Garrity, G.M., Tiedje, J.M., and Cole, J.R. (2007). Naive bayesian classifier for rapid assignment of rRNA sequences into the new bacterial taxonomy. *Appl. Environ. Microbiol.* 73**,** 5261-5267. doi: 10.1128/aem.00062-07.

Zelles, L. (1999). Fatty acid patterns of phospholipids and lipopolysaccharides in the characterisation of microbial communities in soil: a review. *Biol. Fertil. Soils* 29**,** 111-129. doi: 10.1007/s003740050533.

Supplementary Table

Table S1. Primer pairs and PCR conditions used for T-RFLP fingerprinting and amplicon sequencing in this study

Group	Primers	PCR conditions
Bacteria	Ba27f (5'-3')<br>AGA GTT TGA TCM TGG CTC AG<br><br>Ba907r (5'-3')<br>CCG TCA ATT CCT TTG AGT TT	Reverse transcription:<br>30 min 45°C<br><br>PCR: 5 min 95 °C; 13-25 cycles [30 sec 95°C / 30 sec 52°C / 1 min 68°C]; 5 min 68°C
Protists	Euk20f (5'-3')<br>TGC CAG TAG TCA TAT GCT TGT<br><br>Euk519r (5'-3')<br>ACC AGA CTT GYC CTC CAA T	Reverse transcription:<br>20 min 45°C.<br><br>PCR: 5 min 94°C; 25 cycles [30 sec 94°C / 30 sec 52°C / 1 min 70°C]; 5 min 70°C
Fungi	Random hexamers<br><br>nu-SSU-0817-5'-FAM<br>TTAGCATGGAATAATRRAATAGGA<br><br>nu-SSU-1536-3'<br>ATTGCAATGCYCTATCCCCA	Reverse transcription:<br>10 min 25°C, 30 min 60°C, 5 min 80°C<br><br>PCR: 2 min 94°C, 35 cycles [45 sec 94°C / 45 sec 51°C / 1 min 72°C]; 10 min 72°C

Supplementary Figures

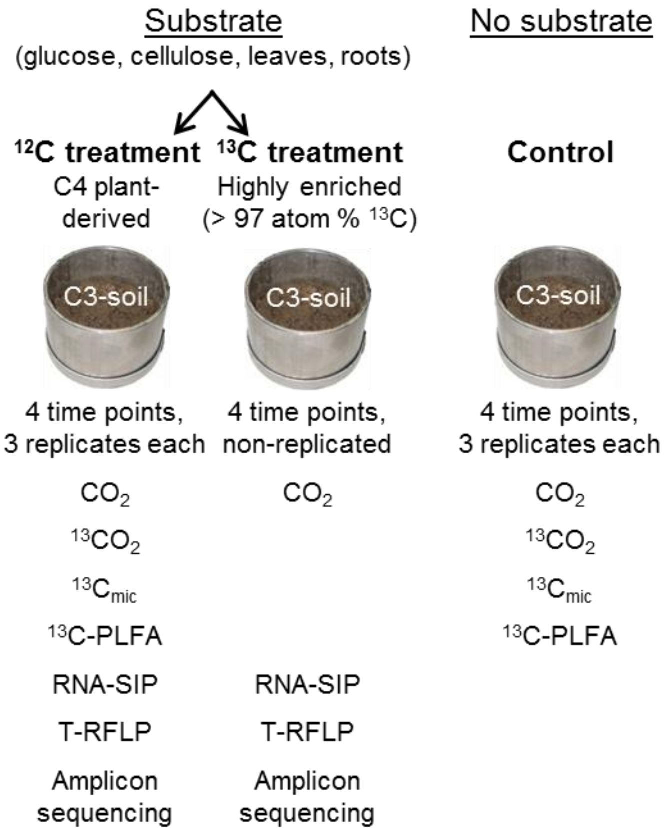

Fig. S1. Scheme of the microcosm setup and downstream workflow of different analyses conducted for the different setups and treatments.

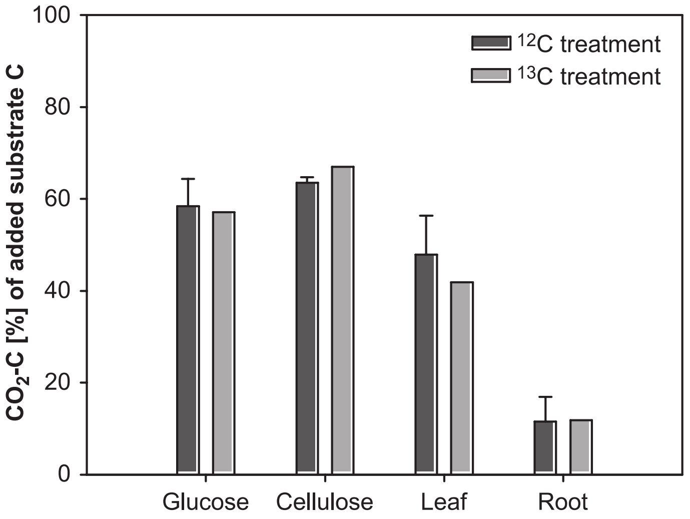

Fig. S2. Cumulative substrate-C mineralized to CO_2 after 32 days of soil incubation.

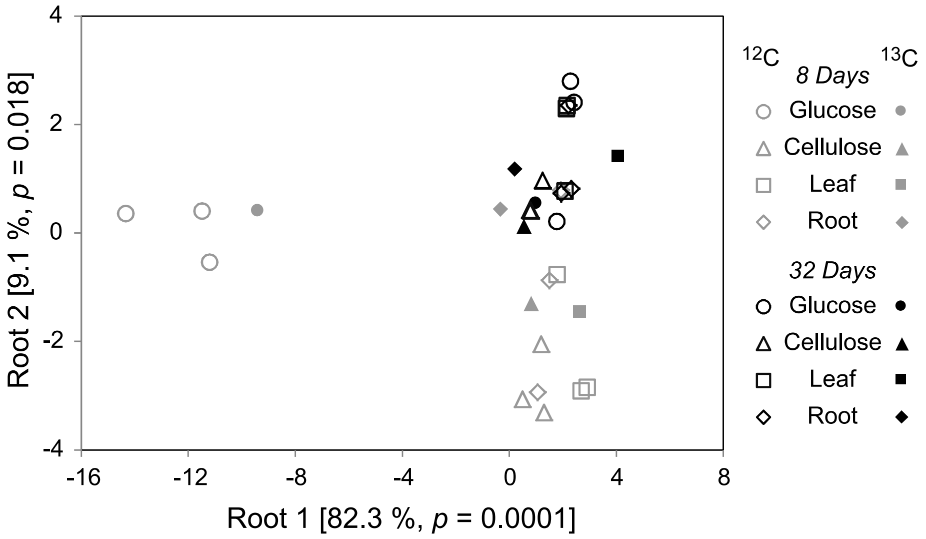

Fig S3. MDS-DFA analysis ordination plot of microbial PLFA profiles in treatments with non-labeled ^{12}C (open symbols) and ^{13}C-labeled (filled symbols) glucose, cellulose, maize leaves and maize roots after 8 and 32 d of incubation. Significance of the whole model was $F_{63,96} = 3.27$; $P < 0.001$. Variance explained by the axes is given as percentage of total variance.

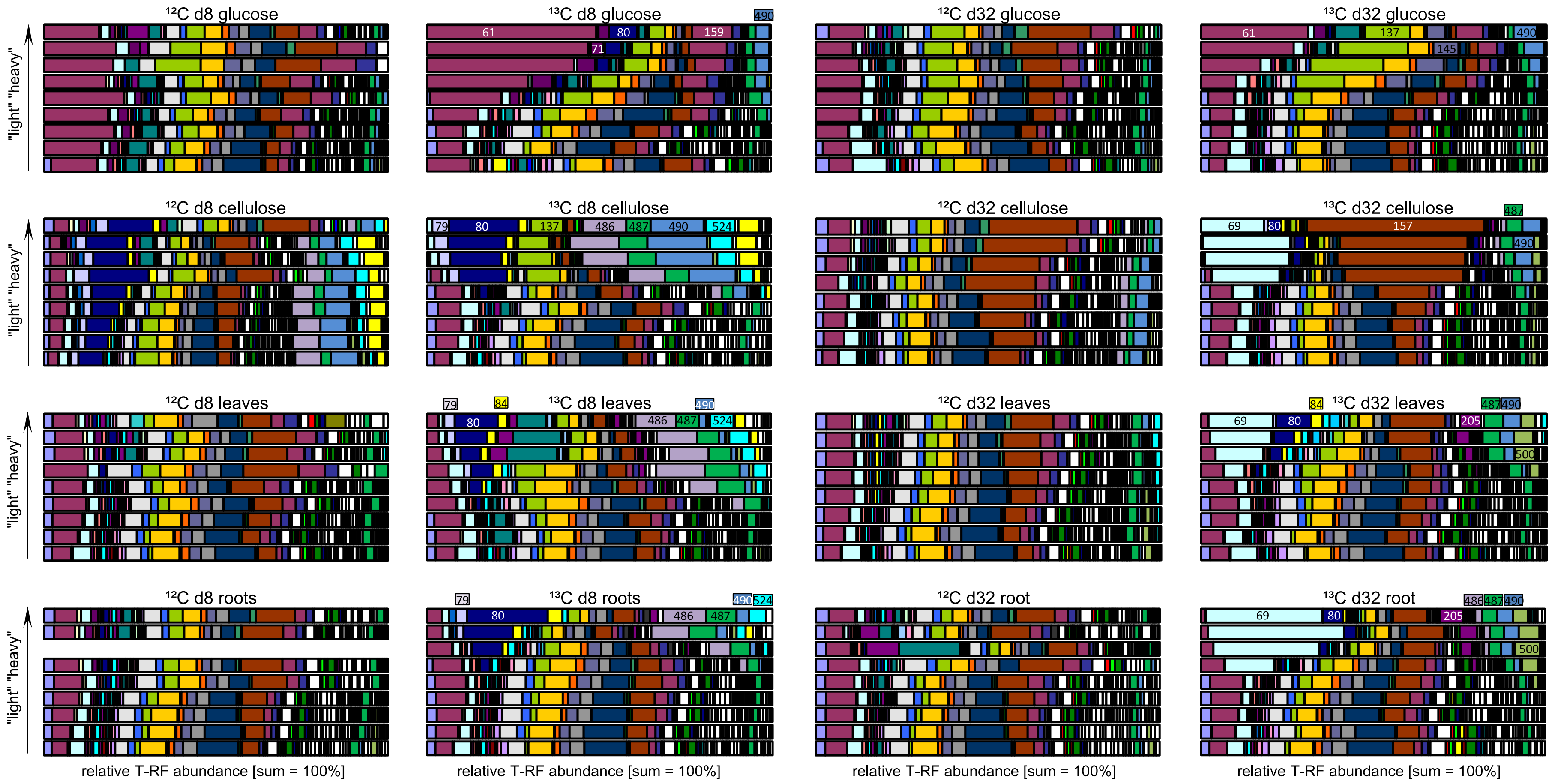

Fig. S4. Bar plots of bacterial 16S rRNA T-RFLP fingerprints from SIP gradients. Arrows indicate rRNA-gradient fractions #2 to #10 of the ^{12}C and ^{13}C treatments. The buoyant density of sequenced fractions #3 and #8 was either ~1.82 or ~1.79 or g ml^{-1} CsTFA, which is typical for heavy and light rRNA, respectively (Lueders et al., 2004). Representative T-RFs identified to represent labeled taxa (mentioned in text) are highlighted [fragment length in bp]. Relative abundance of all T-RFs is 100%.

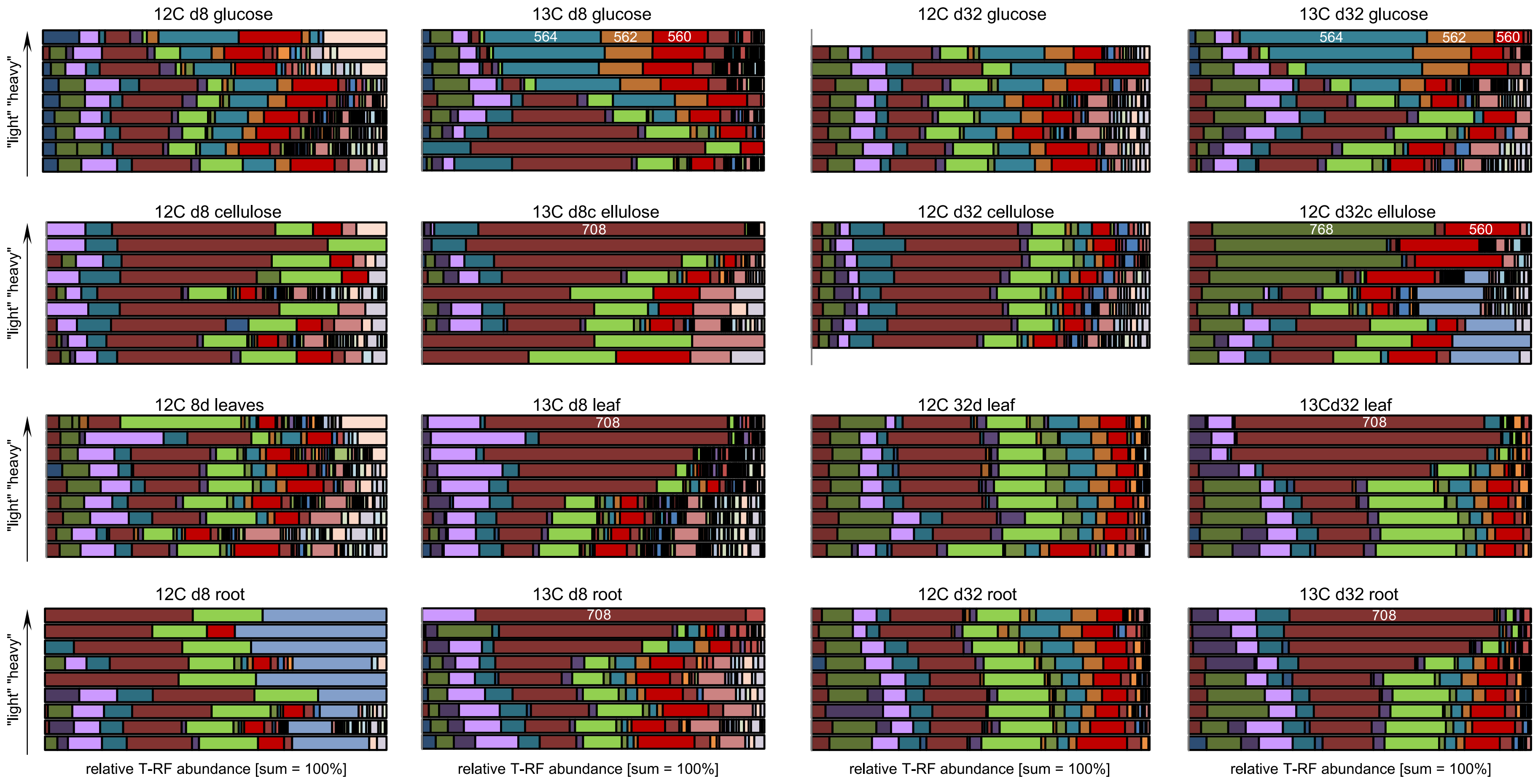

Fig. S5. Bar plots of fungal 18S rRNA T-RFLP fingerprints from SIP gradients. Arrows indicate rRNA-gradient fractions 2 to 10 of the ^{12}C and ^{13}C treatments. The buoyant density of sequenced fractions #3 and #8 was ~1.82 or ~1.79 or g ml^{-1} CsTFA, respectively. Representative T-RFs identified to represent labeled taxa (mentioned in text) are highlighted [fragment length in bp].

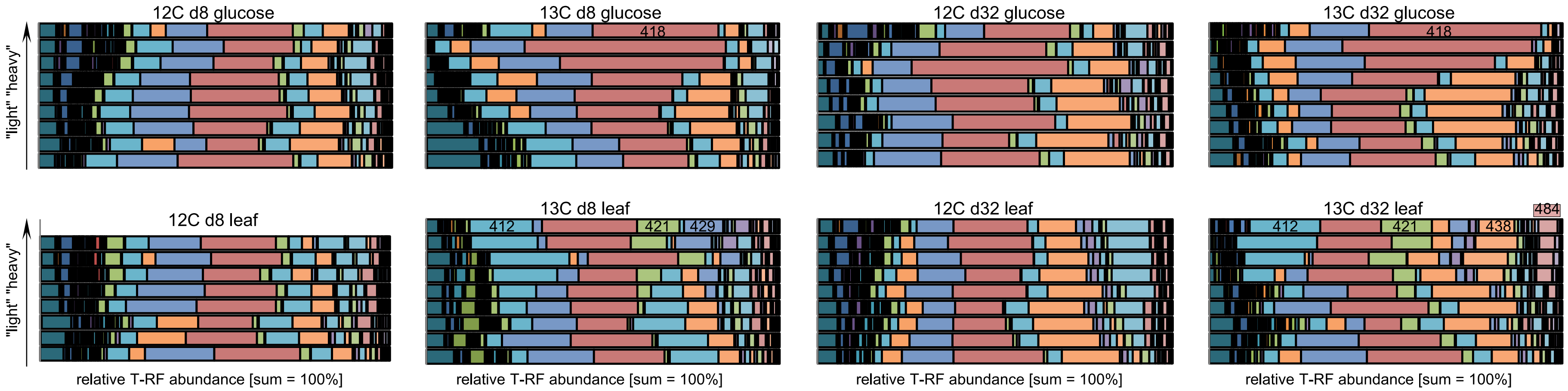

Fig. S6. Bar plots of protist 18S rRNA T-RFLP fingerprints from SIP gradients of the glucose and leaf treatments. Arrows indicate rRNA-gradient fractions 2 to 10 of the ^{12}C and ^{13}C treatments. The buoyant density of sequenced fractions #3 and #8 was ~1.82 or ~1.79 or g ml^{-1} CsTFA, respectively. Selected relevant T-RFs identified to represent labeled taxa and mentioned in the text are highlighted by numbers [fragment length in bp].
